# SCANellome V2: Update of the Primate Anellovirus Reference Sequences Database

**DOI:** 10.3390/v16091349

**Published:** 2024-08-23

**Authors:** Florian Laubscher, Laurent Kaiser, Samuel Cordey

**Affiliations:** 1 Laboratory of Virology, Department of Diagnostics, Geneva University Hospitals & Faculty of Medicine, University of Geneva, 1205 Geneva, Switzerland; florian.laubscher@hcuge.ch (F.L.); laurent.kaiser@hcuge.ch (L.K.); 2 Division of Infectious Diseases, Geneva University Hospitals, 1205 Geneva, Switzerland; 3 Geneva Centre for Emerging Viral Diseases, Geneva University Hospitals, 1205 Geneva, Switzerland

**Keywords:** SCANellome V2, metagenomics, anelloviruses, genomic diversity

## Abstract

Anelloviruses are ubiquitous in humans and represent a major component of the human virome. Its best-known representative is Torque teno virus (i.e., the *Alphatorquevirus* genus), which is considered a potential immunity biomarker. Recent metagenomic investigations revealed not only the extraordinary genomic diversity of anellovirus sequences, but also that co-detection of genera, genotypes, or species seems to be the rule in humans. SCANellome was developed to represent a user-friendly tool to analyze the primate (both human and non-human) anellovirus composition at the genus, species, and genotype level from metagenomics data based on an up-to-date database. This SCANellome update includes >900 additional reference sequences from GenBank. Using a clustering at 90% identity, the FASTA database was updated and generated 134 new representative sequences. Based on ORF1, the analysis of these new sequences indicates the presence of 206 potential new species, including four nonhuman primates, and adds four new non-human primate species which will be the subject of a proposal to the International Committee on Taxonomy of Viruses (ICTV). In addition, SCANellome V2 provides now the user with an interactive up-to-date phylogenetic analysis (of ORF1) to show the distribution among the 12 human and nonhuman primate genera of these new potential species. Finally, the *Anelloviridae* taxonomy was updated to rename species names in binomial format as required by the ICTV.

## 1. Introduction

Anelloviruses are ubiquitous and considered a major component of the human virome. Although no disease has been related to anelloviruses, the term anellome is commonly used to describe the composition of anelloviruses infecting an individual [1]. Anelloviruses are nonenveloped, containing a circular, negative-sense, single-stranded DNA genome ranging from 1.6 to 3.9 kb. Their genomic organization is made up of a large open reading frame (ORF), referred to as ORF1 (coding for the capsid protein, the most conserved protein), and a few smaller ORFs. Of the 34 genera of the *Anelloviridae* family defined by the International Committee on Taxonomy of Viruses (ICTV) (based on the complete ORF1 coding region nucleotide sequences using 69% as a species demarcation threshold) [2], eight have been described in humans, namely the genera *Alphatorquevirus* (commonly called Torque teno virus (TTV)), *Betatorquevirus* (commonly called Torque teno mini virus (TTMV)), *Gammatorquevirus* (commonly called Torque teno midi virus (TTMDV)), *Hetorquevirus* [3,4,5], and more recently *Yodtorquevirus*, *Lamedtorquevirus*, *Memtorquevirus*, and *Samektorquevirus* (last ICTV release).

Next-generation sequencing metagenomic studies not only highlight the impressive genomic diversity of anelloviruses but also that the co-detection of different genera, species, and genotypes within a single individual is the rule rather than the exception [6,7,8]. Investigation of the genomic diversity of anelloviruses in humans is becoming increasingly complex and demanding for bioinformatic experts that are not specialists in the *Anelloviridae* family, requiring them to ensure that the latest classification criteria established by the ICTV are met. Therefore, we previously designed SCANellome, a user-friendly computer tool to evaluate the anellome composition from raw data generated by the Illumina and Nanopore platforms [9]. Indeed, although short-read sequencing platforms (dominated by the Illumina platforms) represent the main contributors to the anellovirus sequences made available in GenBank, more and more investigations now use long-read sequencing technologies such as the Oxford Nanopore platforms, which can be extremely useful for the analysis of circular DNA virus genomes [10].

This SCANellome update includes >900 additional reference sequences from GenBank and has generated a novel FASTA complete ORF1 database using a clustering at 90% identity. The presence of new species based on the ICTV classification criteria were also investigated. In addition, SCANellome V2 now provides the user with a phylogenetic analysis (of ORF1) that includes new species not yet officially classified by the *Anelloviridae* ICTV committee. Finally, the *Anelloviridae* taxonomy was updated in SCANellome V2 to rename species names in binomial format as required by the ICTV [11].

## 2. Materials and Methods

### Scanellome Database 

The database has been updated with GenBank sequences downloaded up to 8 July 2024 and with the keywords for sequence search updated, including new “metagenome_source” and “host” targets (e.g., metagenome_source=”blood metagenome”, host=“Callithrix penicillata”), using a previously described script from https://github.com/Laubscher/Anelloviruses/, accessed on 20 August 2024.

Sequences below the 69% nucleotide identity threshold have been assigned to new potential species. Then a FASTA database was generated using CD-HIT (v4.7) at 90% identity.

An overall phylogeny analysis of the database has been carried out using an amino acid alignment containing one representative sequence for each species using MAFFT for the alignment (v7.520) and MEGA X (v10.0.5) for the tree reconstruction with the Maximum Likelihood method and LG+G+I+F model [12]. According to this analysis, using the ICTV genera taxonomy, including the novel proposed genus *Sadetorquevirus* [13], three species have been reassigned to different genera, one *Omegatorquevirus* to *Betatorquevirus* and two *Hetotorquevirus* to novel genus *Sadetorquevirus*.

Additionally, for each *Alpha*-, *Beta*-, and *Gammatorquevirus* the same phylogeny analysis has been conducted with representative sequences forming a clustering at 70% nucleotide identity using CD-HIT (v4.7). According to this analysis, using the SCANellome group classification, two *Betatorquevirus* and one *Gammatorquevirus* have been moved to an unclassified group.

The header of the FASTA database has been modified to include a new field:

“ICTV=<bool>” with a “True” value if the species is currently approved by the ICTV or a “False” value if not, using the “ICTV Master Species List” (https://ictv.global/msl, accessed on 20 Aug 2024).

All species without ICTV approval have also been renamed with binomial nomenclature. This includes:

- 12 *Alphatorquevirus* using epithet “cero” for Cercopithecidae hosts or “homin” for Hominidae hosts and with number matching former names;
- 144 *Betatorquevirus* using epithet “homini” for Hominidae hosts and with numbers incrementing by the hundreds depending on taxonomy group and by the tens according to their provisional name;
- 50 *Gammatorquevirus* using epithet “homidi” for Hominidae hosts with numbered increments from 16 to 65;
- one *Omegatorquevirus* using epithet “hominid”;
- three *Epsilontorquevirus* using epithet “cebid” for Cebidae hosts or “calli” for Callitrichidae hosts.

All non-ICTV name changes have been tracked in Table S1.

## 3. Results

**Figure 1:**
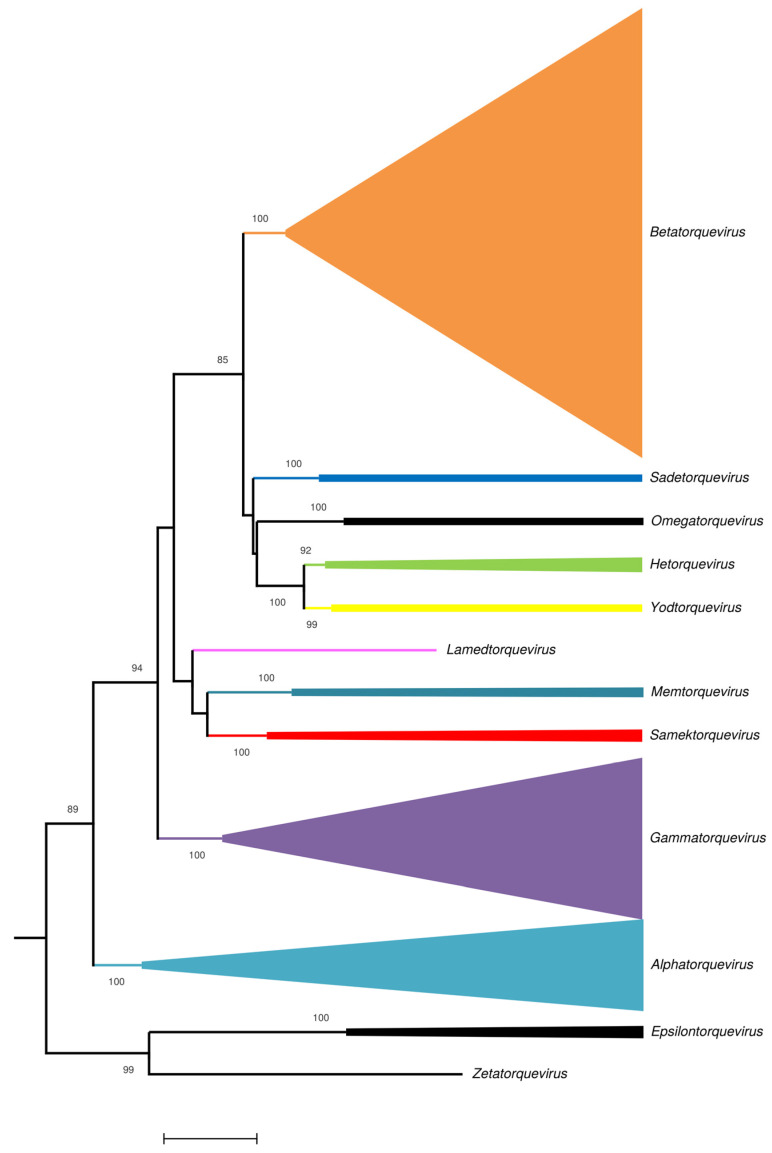
Phylogenetic analysis of anellovirus. Interactive phylogenetic analysis across the 12 primate genera. The Maximum Likelihood tree is based on complete ORF1 nucleotide sequences. By clicking on the name of some genus, the user accesses a more detailed representation describing the species assigned or not within that selected genus. Scale is in number of substitutions per site.

The SCANellome database was updated on 8 July 2024. A total of 916 additional complete ORF1 primate anellovirus sequences were added, generating a database of over 18000 sequences. Using CD-HIT at 90% identity, 134 new representative sequences were generated in SCANellome V2. Furthermore, despite the addition of >900 sequences attributed to viruses with human hosts, there was no change in the number of species infecting humans (neither abolished nor established) in the SCANellome database using the 69% nucleotide identity threshold. Thus, this confirms the provisional species establishment in the SCANellome database (a proposal to the ICTV is in preparation). Table 1 describes the distribution of these additional sequences and potential new species across the 12 different human and nonhuman primate genera. In addition, SCANellome V2 now provides the user with an up-to-date phylogenetic analysis (by ORF1) to show the distribution of these new potential species (Figure 1). Indeed, the user first accesses the “interactive” complete ORF1 phylogenetic tree and can then choose to individually click on each of the three *Alpha*-, *Beta*-, and *Gammatorquevirus* genera to generate a detailed phylogenetic tree highlighting the new species within the selected genus in red. The majority of potential new species are assigned to *Betatorquevirus* (*n* = 144) and *Gammatorquevirus* (*n* = 50).

## 4. Conclusions

SCANellome V2 not only includes a significant number of additional anellovirus sequences, but thanks to these, it allows identification of the presence of more than 200 potential new species. Furthermore, now an up-to-date phylogenetic analysis shows the user the distribution of these new species among the 12 human and nonhuman primate genera. In addition, this updated version allows the user to obtain a nomenclature in binomial format as required by the ICTV in 2023.

## Figures and Tables

**Table 1:** Summary of the number of sequences and species for each genus in the updated (V2) complete ORF1 primate anelloviruses database. The distribution of the potential 210 new species within the 12 genera is also reported. NA: not applicable; * *Hetorquevirus* hominid 7 and 8 in V1 version moved to *Sadetorquevirus* in V2 version; ** moved to *Betatorquevirus*.

Genus	Number of Complete ORF1 Sequences		Representatives 90% Identity		Number of Species		Number of Species without ICTV Approved
	V1	V2	V1	V2	V1	V2	
*Alphatorquevirus*	5444	6096	419	469	36	36	12
*Betatorquevirus*	6659	6820	1856	1908	178	180	144
*Gammatorquevirus*	5534	5617	1376	1393	64	64	50
*Hetorquevirus*	109	69	15	10	7	5	0
*Sadetorquevirus*	NA	41 *	NA	6 *	NA	2 *	0
*Yodtorquevirus*	17	17	2	2	2	2	0
*Lamedtorquevirus*	33	37	5	6	1	1	0
*Memtorquevirus*	55	58	20	22	3	3	0
*Samektorquevirus*	124	128	32	35	4	4	0
*Omegatorquevirus*	3	2 **	3	2 **	3	2	1
*Epsilontorquevirus*	1	10	1	10	1	4	3
*Zetatorquevirus*	1	1	1	1	1	1	0
Total	17,980	18,896	3730	3864	300	304	210

## Data Availability

SCANellome V2 is freely available at: https://laubscher.github.io/Anelloviruses/SCANellome (release date 5 August 2024), the updated complete ORF1 primate anelloviruses database is available at: https://github.com/Laubscher/Anelloviruses/releases (release date 25 July 2024), the source code is available at: https://github.com/Laubscher/SCANellome (release date 5 August 2024), and the dataset is available at: https://doi.org/10.5281/zenodo.7937276 (release date 15 May 2023).

## Supplementary Materials

The following supporting information can be downloaded at: https://www.mdpi.com/article/10.3390/v16091349/s1. Table S1: Summary of the species without ICTV approval; Figure S1: Example of a detailed phylogenetic analysis after the user has selected a specific genus (here *Alphatorquevirus*). Each branch is a representative sequence of a 70% identity cluster. Species assigned or not yet assigned (i.e., potential new species) by the *Anelloviridae* ICTV are represented in black and red, respectively. Scale is in number of substitutions per site.
