# Supplementary figures and images for: SCANellome V2: Update of the Primate Anellovirus Reference Sequences Database
Source: Viruses. 2024 Aug 23;16(9):1349. doi: 10.3390/v16091349 (PMC11435895; doi:10.3390/v16091349)

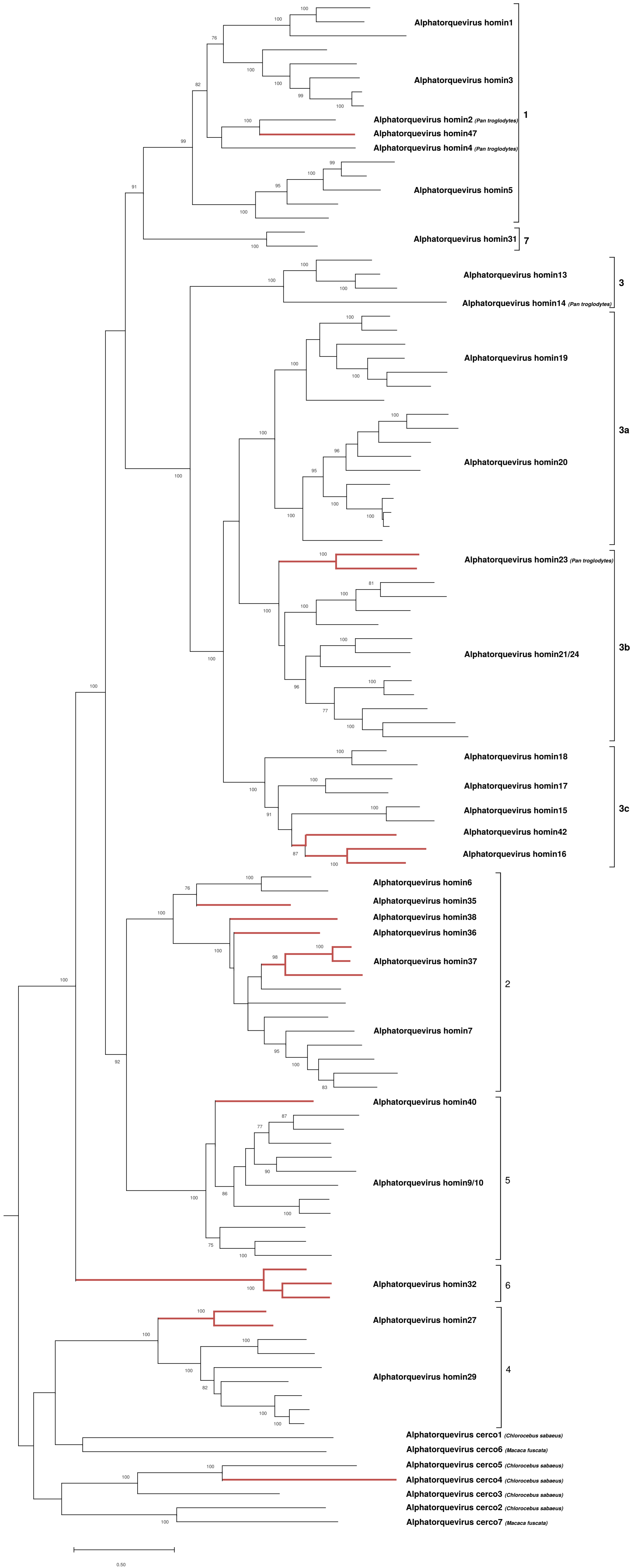

Supplement: Supplementary file 1 [file viruses-16-01349-s001.zip › Figure S1.pdf]
